# Fc *γ* RIIA Genotypes and Its Association with Anti-C1q Autoantibodies in Lupus Nephritis (LN) Patients from Western India

**DOI:** 10.4061/2010/470695

**Published:** 2010-02-09

**Authors:** Vandana Pradhan, Manisha Patwardhan, Anita Nadkarni, Kanjaksha Ghosh

**Affiliations:** National Institute of Immunohaematology, Indian Council of Medical Research, 13th floor, KEM Hospital, Parel, Mumbai 400 012, India

## Abstract

To identify Fc *γ* RIIA genotypes in Systemic Lupus Erythematosus (SLE) patients and their association with anti-C1q antibodies. *Methods*. Fc *γ* RIIA genotyping was done in eighty Indian SLE patients and eighty healthy controls using allele-specific PCR. Anti-C1q antibodies were measured by ELISA. *Results*. LN patients showed higher SLEDAI (6–32) as compared to SLE patients without renal manifestations and had SLEDAI between 6–23. Fc *γ* RIIA polymorphic frequency in SLE patients was R131/H131 (67.5%), R131/R131 (20%) and H131/ H131 (12.5%) as against that of normal population (62.5%, 10%, and 27.5%), respectively. Sixty two patients (77.5%) showed positivity for anti-C1q antibodies. LN patients showed elevated levels of anti-C1q antibodies (258.2 u/ml ± 38.5 U/mL) as compared to SLE patients without nephritis (134.6 ± 24.6 U/mL). Among anti-C1q positive patients, 71% had R131/H131 genotype, 22.6% had R131/R131 and remaining 6.4%, patients had H131/H131 genotype. All anti-C1q positive patients with R131/R131 genotype had elevated levels of anti-C1q antibodies (>100 U/ml), whereas among anti-C1q negative patients, none had R131/R131 genotype. *Conclusion*. This first report on Indian SLE patients supports the hypothesis that Fc *γ* RIIA R131 variant over expression may constitute a susceptibility factor for development of severe SLE manifestations in LN patients.

## 1. Introduction

Genes associated with immune complex clearance, such as Fc receptors for IgG (Fc *γ* R), have been described in the past. Recent interest is focused on possibility that genetically determined polymorphisms in structure and function of Fc *γ* receptors on phagocytic cells may be important in pathogenesis of Systemic Lupus Erythematosus (SLE). An increased association of Fc *γ* RIIA gene polymorphism in SLE patients with renal involvement has been demonstrated. Two different alleles encode for Fc *γ* IIA receptors (R131, H131) (expressed on most leukocytes and on platelets) with differing capacities to bind human IgG2. Antibodies of IgG2 class are efficiently recognized by the H131 allele of Fc *γ* R IIA. In contrast, Fc *γ* R IIA-R131 allele is found to have least binding with IgG2 and is associated with an increase risk for renal disease [1, 2].

C1q is the first component of complement classical pathway. It plays a critical role in clearance of immune complexes and apoptotic bodies from tissues and organs. Anti-C1q antibodies are immunoglobulins that bind to the collagenous portion of C1q via their antigen binding region (Fab). Anti-C1q antibodies have been found in several infectious autoimmune diseases. In SLE, which is a prototypic immune complex disease, anti-C1q antibodies are involved in immunopathogenesis. Anti-C1q antibodies bind to glomerular immune complex deposits, enhancing complement activation and this leads to subsequent tissue injury. Renal deposition of C1q is a characteristic of proliferative lupus nephritis (LN). Anti-C1q antibody titers are increased in LN patients and rising titers are correlated with relapse of nephritis [3–6].

Fc *γ* RIIA R131 phenotype and its association with susceptibility to both infection and autoimmunity have been reported in the literature. The presence of Fc *γ* RIIA R131 allele is associated with susceptibility to the development of glomerulonephritis in SLE. There are sporadic reports available on presence of Fc *γ* RIIA R131 variant and its association with an increase risk of renal disease in SLE patients with anti-C1q antibodies [7–10]. The study was designed to find out Fc *γ* RIIA genotype frequencies in Indian SLE patients and normal healthy controls. We also tried to evaluate the association of Fc *γ* RIIA genotypes, anti-C1q antibody positivity with clinical manifestation of the patient with LN.

## 2. Material and Methods

### 2.1. Subjects

Eighty SLE patients, including 53 renal biopsy-proven cases of LN and 27 cases of SLE without clinical evidence of nephritis (consistently normal renal function) were selected for this study over a period of 2 years. The details of clinical, histopathological, and laboratory findings were recorded. This retrospective study was carried out after obtaining the requisite Ethics Committee permission. SLE patients were diagnosed based on the American College of Rheumatology ACR criteria. SLE disease activity was assessed in all these patients by the SLE Disease Activity Index (SLEDAI). All the patients were in active stage of disease and were untreated when included in the study [11, 12]. 

Patients had no history of taking any drugs such as hydralazine and propylthiouracil. Pregnant or postmenopausal women were excluded. Eighty age matched healthy subjects were used as normal controls. Blood was collected after obtaining informed consent and sera were stored in aliquots at −80°C until tested. Renal biopsies were examined by light microscopy with hematoxylin, eosin and periodic Schiff (PAS) staining and by immunofluorescence microscopy using anti-IgG, anti-IgM, anti-IgA, anti-C3, anti-C4, and anti-fibrinogen fluorescein isothiocyanate conjugate (FITC). In LN patients the renal histology was classified according to WHO criteria [13].

### 2.2. Methods

**Figure 1:**
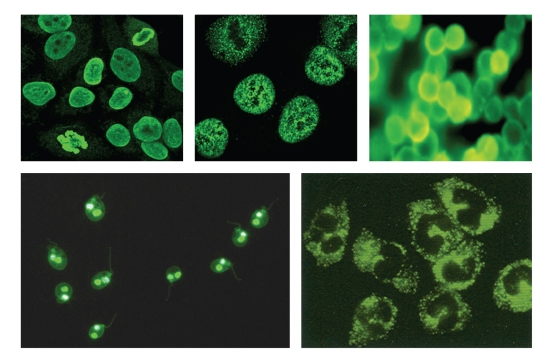
Classical Patterns of Anti-Nuclear Antibodies (Row 1: Left to Right. Nuclear Homogenous, Coarse Speckled and Peripheral Pattern); Row 2: Left to right. Anti-double stranded antibodies (anti-dsDNA), Anti-Neutrophil Cytoplasmic antibodies (ANCA).

Anti-C1q antibodies were detected using anti-C1q EIA kit (Binding Site, UK). Levels of anti-C1q antibodies below 8 U/mL were taken as negative while levels above 8 U/mL were considered as positive. The measuring range varied between 1.23–100 U/mL. Values greater than this range were interpreted as >100 U/mL. Anti-nuclear antibodies (ANA) were tested using Bio Rad kit where HEP-2 cell line was used as a substrate. Results were recorded using a fluorescence microscope (Nikon, Optiphot II). Confirmation of unusual and rare ANA patterns was done using a Confocal Laser Scanning Microscope (Karl, Zeiss, LSM -510). Anti-Neutrophil cytoplasmic antibodies (ANCA) and anti-double stranded DNA (Anti-dsDNA) were detected using Euroimmune, Lubeck kit. (Figure 1) Anti-Histone antibodies were detected by ELISA. 

**Figure 2:**
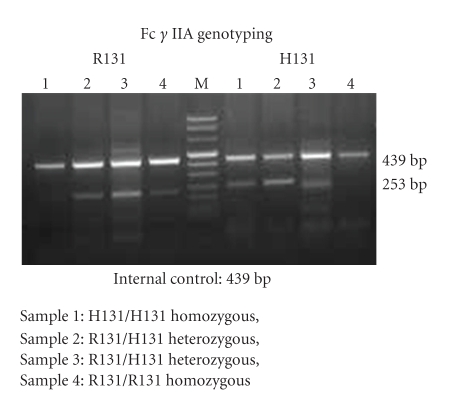
Agarose gel electrophoresis Fc *γ* IIA polymorphism.

The genomic DNA was extracted using standard protocol [14]. Fc *γ* RIIA genotyping was performed using 25 *μ*l PCR reaction containing 100 ng of genomic DNA, 15 mM MgCl_2_, 200 *μ*M dNTP, and 0.5 U of *Taq* polymerase (Ampli Taq Gold, Perkin Elmer, CA); 0.5 *μ*M H131-specific sense primer (5′-ATC CCA GAA ATT CTC CCA-3′) or 0.5 *μ*M R131-specific sense primer (5′-ATC CCA GAA ATT CTC CCG-3′) with 0.5 *μ*M common antisense primer (5′-CAA TTT TGC TGC TAT GGG C-3′) was used. Human growth hormone (HGH) was used as internal control. The forward primer was HGH-1(5′-CAG TGC CTT CCC AAC CAT TCC CTT A-3′) and reverse primer was HGH-2 (5′-ATC CAC TCA CGG ATT TCT GTT GTG TTT C-3′). The PCR conditions were as follows: The initial denaturation was carried out at 95°C for 5 min. Next 10 cycles consisted of 95°C-1 min, 57°C-2 min, and 72°C-1 min; and then to enhance the sensitivity, 22 cycles of 95°C-1 min, 57°C-2 min, and 72°C-1 min were carried out. Final extension was carried out at 72°C-5 min. Amplified product was later on run on 1.5% agarose gel [15] (Figure 2).

### 2.3. Statistical Analysis

Continuous variables are expressed as mean ± SD. Pairs of groups were compared using student's “*t*” test for normally distributed continuous distribution. The “*X* ^2^” test was used for the categorical variables as needed. Statistical significance was set at *P* ≤ .05. 

### 2.4. Results

Of 80 female patients, 53 patients (66.3%) were LN and the remaining 27 patients (33.7%) were SLE without nephritis. Renal histopathology of LN patients revealed that 34 patients (64.2%) were DPGN (Class IV), 16 (30.2%) were FPGN (Type III), and 3 (5.7%) were MPGN (Type, V). LN patients showed slightly higher SLEDAI (6 to 32) as compared to SLE patients without renal involvement (6 to 23).  Table 1 gives demographic details of anti-C1q positive and anti-C1q negative groups. Out of 80 patients tested for anti-C1q antibodies, 62 patients (77.5%) had anti-C1q antibodies of which 52 (76.4%) had anti-C1q values >100 U/mL. Elevated levels of anti-C1q antibodies 258.2 U/mL ± 38.5 U/mL were detected in LN patients as compared to 134.6 ± 24.6 U/mL in SLE without nephritis. Eighteen patients (22.5%) showed absence of anti-C1q antibodies. 

Details of Fc *γ* IIA genotyping in SLE patients and normal controls are given in Table 2. Among normal individuals, 10% of cases showed homozygosity for R131 allele (R131/R131) while 27.5% were homozygous for H131 allele (H131/H131) and the remaining 62.5% showed heterozygosity for both the alleles (R131/H131). Among SLE patients 16 (20%) showed homozygosity for R131 allele (R131/R131) while 10 (12.5%) patients were homozygous for H131 allele (H131/H131) and the remaining 54 patients (67.5%) showed heterozygosity for both the alleles (R131/H131). Comparison of Fc *γ* IIA genotyping with anti-C1q antibodies was carried out in 62 anti-C1q positive patients. Fourteen patients (22.6%) homozygous for R131 allele showed elevated anti-C1q antibody levels (>100 U/mL). Among 44 patients showing heterozygosity for R131/H131, 35 (79.6%) showed elevated anti-C1q antibody levels (>100 U/mL) and remaining 9 patients (20.4%) had equivocal values for anti-C1q antibodies. H131/H131 homozygosity was found in only 4 patients (6.4%) that were positive for anti-C1q antibodies. It was observed that among 18 anti-C1q negative patients, none had R131/R131 genotype, 14 patients (77.8%) were R131/H131 heterozygous, and 4 patients (22.2%) had H131/H131 homozygous genotype. 

Table 3 gives distribution of Fc *γ* RIIA genotypes and SLEDAI scores in 80 SLE patients. It was observed that 28 patients (35%) had severe disease activity (SLEDAI >18), 40 patients (50%) had moderate disease activity (SLEDAI 8 to 18), and 12 patients had mild disease activity (SLEDAI <8). It was observed that 10/16 patients (62.5%) having Fc *γ*  IIA R131/R131 genotype had severe disease activity, whereas 28/56 patients (50%) having Fc *γ* IIA R131/H131 genotype had moderate disease.  Table 4 gives the correlation of Fc *γ* IIA polymorphism with clinical characteristics, anti-C1q antibodies, and other autoantibodies.

## 3. Discussion

Receptors for the Fc domains of IgG (Fc *γ* R) play a critical role in linking humoral and cellular immune responses. Different Fc *γ* receptor genes may contribute to infectious and immune related diseases in various ethnic populations. Polymorphisms in Fc *γ* R gene mainly Fc *γ* R IIA, IIB, IIIA, and IIIB have been identified as genetic factors influencing susceptibility to disease. Activated and inhibitory Fc *γ* Rs seem to play an important role in pathogenesis of SLE, initiation of autoimmunity, subsequent development of inflammatory lesions, and finally immune complex (IC) clearance mechanisms [10].

This is the first report from India identifying Fc *γ* RIIA genotype frequencies and their association with anti-C1q antibodies in LN and SLE without nephritis, patients. Anti-C1q antibodies have been found to correlate with renal disease activity in SLE patients and consequently rising titers may be predictive for ensuring relapses or flares of LN [16, 17]. Lee and Madaio had reported an importance of anti-C1q antibody detection as a new measure for renal involvement [18]. For LN, the critical issues include identifying these patients at risk for flare, progressive nephritis and development of end stage renal disease. A higher prevalence of anti-C1q antibodies has been reported in LN [18]. It had also been shown that the absence of anti-C1q antibodies excludes a diagnosis of LN, whereas increases in levels may predict renal flares [19, 20].

Bazilio et al. had reported an apparent skewing towards Fc *γ* IIA R 131 genotype in Brazilian SLE patients with class III and IV types as compared to controls where low affinity Fc *γ* IIA R 131 variant is found not only associated with proliferative GN but also with a more intense IgG2 glomerular deposition [8]. Fang et al. had reported the prevalence of anti-C1q antibodies in DPGN cases of LN. It was reported that IgG2 subclass of anti-C1q antibodies might be pathogenic and IgG3 subclass of anti-C1q might be more specific bio-marker for monitoring disease activity [21]. Maura et al. had reported a positive correlation between high titres of anti-C1q antibodies, and SLEDAI scores in Brazilian SLE patients and had shown an association of anti-C1q antibodies with LN [22]. Our study also supported this finding. Trendelenburg et al. reported a very high incidence (97.2%) of anti-C1q antibodies in patients with proliferative LN compared with 35% of SLE patients with inactive LN and 25% in SLE patients without nephritis, suggesting that a negative test result for anti-C1q antibodies almost excludes active nephritis indicating a pathogenic role of anti-C1q antibodies [16]. The pathogenic role of IgG2 subclass of anti-C1q antibodies may relate to impaired IC clearance by mononuclear phagocyte system.

In a Korean study it was shown that LN was less frequent in Fc *γ* R IIA R131/H131 heterozygous and H131/H131 homozygous patients as compared to R131/R131 homozygous patients. Studies on Dutch and African-American SLE patients had further strengthened this hypothesis of association of Fc *γ* R IIA R131/R131 homozygosity with LN [8]. Norsworthy et al. had also reported similar findings in Caucasoid patients where a close association between Fc *γ* R IIA R131 variant, anti-C1q antibodies and glomerulonephritis was reported. This may be due to the failure of IC clearance of anti-C1q autoantibodies that are pathogenic in nature [9]. Similarly, findings from our study also support that Fc *γ* R IIA R131 constitutes a susceptibility factor for the development of severe SLE that affects the kidney. 

## Figures and Tables

**Table 1:** Demographic data of anti-C1q positive and anti-C1q negative patients.

Parameters	Anti-C1q positives (*n* = 62)	Anti-C1q negatives (*n* = 18)
Mean age in years		
Range	18–45	16–38
(Mean ± SD)	30.6 ± 10.8	27.5 ± 9.5

SLE duration in months		
Range	6–18	6–15
(Mean ± SD)	8.6 ± 3.2	9.0 ± 2.5

SLEDAI		
Range	6–32	6–23
(Mean ± SD)	(6.0 ± 6.8)	(5.8 ± 6.5)

Lupus Nephritis (*n* = 53)	47 (88.7%)	6 (11.3%)
FPGN (Type III) (*n* = 16)	14	2
DPGN (Type IV) (*n* = 34)	30	4
MPGN (Type V) (*n* = 3)	3	0

SLE without nephritis (*n* = 27)	15 (55.5%)	12 (44.4%)

**Table 2:** Fc *γ* RIIA genotyping in Indian SLE patients and normal controls.

Population	R131	H131	R131/R131	R131/H131	H131/H131
Normal Controls	0.4	0.6	8	50	22
(*n* = 80)			(10%)	(62.5%)	(27.5%)
All SLE	0.55	0.45	16*	54	10*
(*n* = 80)			(20%)	(67.5%)	(12.5%)
Lupus Nephritis	0.55	0.45	10	38	5*
(*n* = 53)			(18.9%)	(71.7%)	(9.4%)
SLE without	0.52	0.48	6	16	5
nephritis (*n* = 27)			(22.2%)	(59.3%)	(9.4%)
Anti-C1q positives	0.57	0.43	14	44	4
(*n* = 62)			(22.6%)	(71.0%)	(6.4%)
Anti-C1q	0.42	0.58	—	14	4
Negatives (*n* = 18)			(0%)	(77.8%)	(22.2%)

(*Significant “*P*” value: *P* < .05).

**Table 3:** Distribution of Fc *γ* RIIA genotypes and SLEDAI in SLE patients.

Fc *γ* IIA genotype	Mild (<8)	Moderate (8–18)	Severe (>18)
R131/R131 (*n* = 16)	2	4	10
R131/H131 (*n* = 54)	10	26	18
H131/H131 (*n* = 10)	0	10	0
Total (*n* = 80)	12 (15%)	40 (50%)	28 (35%)

**Table 4:** Correlation of Fc *γ* RIIA polymorphism with clinical profile and autoantibodies in SLE patients.

Organ involvement	Total SLE (*n* = 80)	R131/R131 (*n* = 16)	R131/H131 (*n* = 54)	H131/H131 (*n* = 10)
Rash	50	14	32	4
Photosensitivity	42	14	26	2
Oral ulcers	18	6	5	1
Arthritis	45	12	31	2
Serositis	8	5	3	0
Renal	53	10	38	5
Hematolgical	10	3	7	0
Neurological	5	2	3	0

Autoantibodies				

ANA	80	16	54	10
Anti-dsDNA	72	14	52	6
ANCA	16	10	6	0
Anti-Histone	28	18	8	2
Anti-C1q	68	12	42	4

## References

[1] Reefman E, Limburg PC, Kallenberg CGM, Bijl M (2005). Fc*γ* receptor in the initiation and progression of systemic lupus erythematosus. *Annals of the New York Academy of Sciences*.

[2] Magnusson V, Johanneson B, Lima G, Odeberg J, Alarcón-Segovia D, Alarcón-Riquelme ME (2004). Both risk alleles for Fc*γ*RIIA and Fc*γ*RIIIA are susceptibility factors for SLE: a unifying hypothesis. *Genes and Immunity*.

[3] Shoenfeld Y, Szyper-Kravitz M, Witte T (2007). Autoantibodies against protective molecules—C1q, C-reactive protein, serum amyloid P, mannose-binding lectin, and apolipoprotein A1: prevalence in systemic lupus erythematosus. *Annals of the New York Academy of Sciences*.

[4] Kallenberg CGM (2008). Anti-C1q autoantibodies. *Autoimmunity Reviews*.

[5] Martens HA, Zuurman MW, de Lange AHM (2009). Analysis of C1q polymorphisms suggests association with systemic lupus erythematosus, serum C1q and CH50 levels and disease severity. *Annals of the Rheumatic Diseases*.

[6] Marto N, Bertolaccini ML, Calabuig E, Hughes GRV, Khamashta MA (2005). Anti-C1q antibodies in nephritis: correlation between titres and renal disease activity and positive predictive value in systemic lupus erythematosus. *Annals of the Rheumatic Diseases*.

[7] Haseley LA, Wisnieski JJ, Denburg MR (1997). Antibodies to C1q in systemic lupus erythematosus: characteristics and relation to Fc*γ*RIIA alleles. *Kidney International*.

[8] Bazilio AP, Viana VST, Toledo R, Woronik V, Bonfá E, Monteiro RC (2004). Fc*γ*RIIa polymorphism: a susceptibility factor for immune complex-mediated lupus nephritis in Brazilian patients. *Nephrology Dialysis Transplantation*.

[9] Norsworthy P, Theodoridis E, Botto M (1999). Overrepresentation of the Fc*γ* receptor type IIA R131/R131 genotype in Caucasoid systemic lupus erythematosus patients with autoantibodies to C1q and glomerulonephritis. *Arthritis and Rheumatism*.

[10] Manger K, Repp R, Jansen M (2002). Fc*γ* receptor IIa, IIIa, and IIIb polymorphisms in German patients with systemic lupus erythematosus: association with clinical symptoms. *Annals of the Rheumatic Diseases*.

[11] Hochberg MC (1997). Updating the American College of Rheumatology revised criteria for the classification of systemic lupus erythematosus. *Arthritis and Rheumatism*.

[12] Bombardier C, Gladman DD, Urowitz MB, Caron D, Chang CH (1992). Derivation of the SLEDAI: a disease activity index for lupus patients. *Arthritis and Rheumatism*.

[13] Weening JJ, D’Agati VD, Schwartz MM (2004). The classification of glomerulonephritis in systemic lupus erythematosus revisited. *Journal of the American Society of Nephrology*.

[14] Elles R, Old JM Laboratory procedures for DNA analysis: WHO training course in standard techniques and advanced methodologies for the control of hereditary anemia’s.

[15] Flesch BK, Bauer F, Neppert J (1998). Rapid typing of the human Fc*γ* receptor IIA polymorphism by polymerase chain reaction amplification with allele-specific primers. *Transfusion*.

[16] Trendelenburg M, Lopez-Trascasa M, Potlukova E (2006). High prevalence of anti-C1q antibodies in biopsy-proven active lupus nephritis. *Nephrology Dialysis Transplantation*.

[17] Horák P, Heřmanová Z, Zadražil J (2006). C1q complement component and antibodies reflect SLE activity and kidney involvement. *Clinical Rheumatology*.

[18] Lee IJ, Madaio MP (2008). Search for useful tools: biomarkers in lupus nephritis. *Future Rheumatology*.

[19] Moroni G, Trendelenburg M, Del Papa N (2001). Anti-C1q antibodies may help in diagnosing a renal flare in lupus nephritis. *American Journal of Kidney Diseases*.

[20] Horváth L, Cziriják L, Fekete B (2001). High levels of antibodies agains C1q are associated with disease activity and nephritis but not with other organ manifestations in SLE patients. *Clinical and Experimental Rheumatology*.

[21] Fang Q-Y, Yu F, Tan Y (2009). Anti-C1q antibodies and IgG subclass distribution in sera from Chinese patients with lupus nephritis. *Nephrology Dialysis Transplantation*.

[22] Moura CG, Lima I, Barbosa L (2009). Anti-C1q antibodies: association with nephritis and disease activity in systemic lupus erythematosus. *Journal of Clinical Laboratory Analysis*.

